# Multiple Insecticide Resistance in the Malaria Vector *Anopheles funestus* from Northern Cameroon Is Mediated by Metabolic Resistance Alongside Potential Target Site Insensitivity Mutations

**DOI:** 10.1371/journal.pone.0163261

**Published:** 2016-10-10

**Authors:** Benjamin D. Menze, Jacob M. Riveron, Sulaiman S. Ibrahim, Helen Irving, Christophe Antonio-Nkondjio, Parfait H. Awono-Ambene, Charles S. Wondji

**Affiliations:** 1 Vector Biology Department, Liverpool School of Tropical Medicine, Liverpool, Merseyside, United Kingdom; 2 LSTM/OCEAC Research Unit. Organisation de Coordination pour la lutte contre les Endémies en Afrique Centrale, Yaoundé, Centre Region, Cameroon; 3 Biochemistry Department, Bayero University Kano, Kano, Kano State, Nigeria; University of Crete, GREECE

## Abstract

**Background:**

Despite the recent progress in establishing the patterns of insecticide resistance in the major malaria vector *Anopheles funestus*, Central African populations of this species remain largely uncharacterised. To bridge this important gap and facilitate the implementation of suitable control strategies against this vector, we characterised the resistance patterns of *An*. *funestus* population from northern Cameroon.

**Methods and Findings:**

Collection of indoor-resting female mosquitoes in Gounougou (northern Cameroon) in 2012 and 2015 revealed a predominance of *An*. *funestus* during dry season. WHO bioassays performed using F_1_ *An*. *funestus* revealed that the population was multiple resistant to several insecticide classes including pyrethroids (permethrin, deltamethrin, lambda-cyhalothrin and etofenprox), carbamates (bendiocarb) and organochlorines (DDT and dieldrin). However, a full susceptibility was observed against the organophosphate malathion. Bioassays performed with 2015 collection revealed that resistance against pyrethroids and DDT is increasing. PBO synergist assays revealed a significant recovery of susceptibility for all pyrethroids but less for DDT. Analysis of the polymorphism of a portion of the voltage-gated sodium channel gene (VGSC) revealed the absence of the L1014F/S *kdr* mutation but identified 3 novel amino acid changes I877L, V881L and A1007S. However, no association was established between VGSC polymorphism and pyrethroid/DDT resistance. The DDT resistant 119F-GSTe2 allele (52%) and the dieldrin resistant 296S-RDL allele (45%) were detected in Gounougou. Temporal analysis between 2006, 2012 and 2015 collections revealed that the 119F-GSTe2 allele was relatively stable whereas a significant decrease is observed for 296S-RDL allele.

**Conclusion:**

This multiple resistance coupled with the temporal increased in resistance intensity highlights the need to take urgent measures to prolong the efficacy of current insecticide-based interventions against *An*. *funestus* in this African region.

## Background

Recent successes in reducing malaria burden across Sub-Sahara African is largely attributed to insecticide-based interventions such as Long Lasting Insecticide Nets (LLINs) and Indoor Residual Spraying (IRS) [1]. Maintaining the effectiveness of such control tools will require tackling the growing problem of insecticide resistance in malaria vectors across the continent [2]. Characterisation of resistance profiles and elucidation of underlying resistance mechanisms for malaria vectors is a prerequisite to develop suitable resistance management tools. Unfortunately such knowledge remains limited in many parts of Africa particularly for a major malaria vectors such as *An*. *funestus*. This is the case for the Central African region, notably in Cameroon, where little is currently known about the resistance status of *An*. *funestus* populations to the main insecticide classes. In Cameroon, malaria is responsible for 30–35% of the total deaths annually, 45% of morbidity cases and around 30% of the population has at least one episode of malaria each year [3]. The major malaria vectors across Cameroon are *Anopheles gambiae s*.*s*., *Anopheles coluzzii*, *Anopheles arabiensis* and *Anopheles funestus s*.*s*. [4]. Cases of insecticide resistance have been mainly documented for *An*. *gambiae s*.*l*., in Cameroon [5–8] with little information available for *An*. *funestus s*.*s*. despite the high *Plasmodium* infection rate of this species which sometimes even surpasses that of *An*. *gambiae* [9]. The available information on *An*. *funestus* is limited to cases of DDT and dieldrin resistance documented in the northern part of the Cameroon [10], with the detection of two resistance markers: the L119F-GSTe2 DDT resistance marker [11] and the A296S dieldrin resistance marker [10]. Importantly, the susceptibility status towards the pyrethroids, the only insecticide class recommended for bed net impregnation remains uncharacterised. Resistance to different classes of insecticides such as pyrethroids, carbamates and DDT are increasingly reported for *An*. *funestus* populations across Africa although with contrasting patterns [12, 13]. *An*. *funestus* populations from southern Africa are highly resistant to pyrethroids and carbamates [13–16] whereas populations from East Africa remain fully susceptible to carbamates but are resistant to pyrethroids and DDT [12, 17–19]. On the other hand, populations from West Africa have been reported to be resistant to pyrethroids, DDT and moderately resistant to carbamates [10, 20, 21]. To understand the dynamics of these resistance profiles throughout the continent, it is necessary to investigate the susceptibility status of this important vector in the Central Africa region.

Insecticide resistance cases observed in *An*. *funestus* populations so far are mainly caused by metabolic resistance mechanisms either for pyrethroids, carbamates or DDT as neither the L1014F/S *kdr* mutation in the VGSC nor the G119S *Ace-1* mutation has been detected in this species [22]. P450 genes have been found to be associated with pyrethroid resistance [23–25] whereas, glutathione-S transferases notably the GSTe2 gene have been shown to confer DDT resistance [11].

Cameroon is currently scaling up its malaria control program through LLINs with ongoing free distribution of nets to households. It is crucial that information on the susceptibility of malaria vectors to the major public health insecticides and the underlying mechanisms of resistance are properly investigated. This will adequately inform control programs of the most suitable insecticides to use and facilitate the design of appropriate resistance management strategies.

In this study, we report the resistance status of *An*. *funestus* population from northern Cameroon to several insecticides used in public health and also explore the underlying molecular basis of the resistance.

## Methods

### Study site

Adult Mosquitoes were collected from the locality of Gounougou (9° 03′ 00″N, 13°43′59″ E) in northern Cameroon. The area is surrounded by the Benoué River with a major hydroelectric dam supplying electricity to the region and also providing water to irrigate about 15,000 hectares of crops downstream. In some areas, the river is surrounded by vegetation. In addition, the presence of a large rice field makes the area suitable for the development of the major malaria vector *An*. *funestus* by providing permanent breeding sites. The area is also characterised by the presence of cotton fields with indiscriminate use of insecticides by local farmers including pyrethroids and DDT [26].

### Mosquito collections

Indoor resting, blood fed female *An*. *funestus* were collected on the walls and on the roof of different houses across the village. The collection was carried out between 6:00 and 11:00 AM following a verbal consent from the chief of the district and the household owners. This was done between the months of December 2011 to January 2012, at the beginning of the dry season. Subsequently, another collection was performed in January 2015 to monitor the dynamics of the resistance in this location. In addition *An*. *funestus* mosquitoes collected in 2006 in the same location, described previously in [10], were also used during this study for TaqMan genotyping of some resistance markers. However, unless specifically mentioned, the samples mentioned throughout the study will be those from 2012. Mosquitoes were collected using aspirators and were kept in paper cups in a cool place prior to transport to the insectary at OCEAC. The forced-egg laying method was used to generate F_1_ individuals for bioassays as described previously [12]. Briefly, each fully gravid female was transferred into a 1.5ml micro-centrifuge tube containing a slightly wet filter paper, and allowed to lay eggs. Eggs were stored at room temperature for up to 3 days and then transported to the Liverpool School of Tropical Medicine (LSTM), UK (under the LSTM import license from DEFRA). Eggs were allowed to hatch in small cup and later transferred to larvae bowl for rearing to the adult stage. Mosquitoes were maintained under standard insectary conditions with a temperature of ~25°C, relative humidity of 70–80% and light:dark cycles of 12:00 hours each. Adults were maintained on 10% sucrose.

#### Species identification

All F_0_ female mosquitoes that laid eggs were morphologically identified as *An*. *funestus* using a morphological key [27]. Genomic DNA was extracted from field collected mosquitoes using the Livak protocol [28]. A cocktail PCR was performed to confirm the identity of the *An*. *funestus* species as previously described [29].

### Insecticide susceptibility bioassays

Susceptibility assays were performed according to WHO protocol [30] using 2–5 day-old mosquitoes. Four replicates of around 20–25 mosquitoes per tube were exposed to insecticide-impregnated filter papers for 1h and then transferred to a clean holding tube supplied with 10% sugar. Mortalities were determined 24h after exposure. The following insecticides were tested for 2012: the pyrethroids permethrin (0.75%, type I), deltamethrin (0.05%, type II), lambda-cyhalothrin (0.05%, type II) and etofenprox (0.05%, ether pyrethroid); the carbamate bendiocarb (0.01%); the organophosphates malathion (5%) and fenitrothion (5%); the organochlorines DDT (4%) and dieldrin (4%). For the 2015 collection, permethrin, deltamethrin, bendiocarb, DDT and malathion were tested. Susceptibility status was established as follows: between 98–100% mortality the population was considered susceptible, between 90–97% mortality, resistance suspected and need confirmation whereas less than 90% was considered resistant [31]). The mosquitoes alive after exposure were kept in -80°C and the dead individuals were stored in silica gel.

#### PBO synergist assays

For the 2012 collection, adult mosquitoes were pre-exposed to 4% piperonyl butoxide (PBO) for one hour, then exposed to either DDT, permethrin, deltamethrin, lambda-cyhalothrin or etofenprox for another one hour. After 24 hours the mortality was assessed and compared with results obtained without pre-exposure to PBO in order to assess any possible involvement of cytochrome P450-mediated resistance.

### Analysis of the voltage-gated sodium channel (VGSC) gene

A portion of the VGSC (917bp) spanning the entire intron 19 and the exon 20 containing the 1014 mutation associated with knockdown resistance (*kdr*) in *An*. *gambiae* [32, 33] was amplified and sequenced. The amplification and the sequencing were done using 5 dead and 5 alive mosquitoes exposed to DDT and permethrin from 2012 collection. The sequences for alive and dead for each insecticide were compared in order to investigate the correlation between the polymorphism pattern of this gene and the resistance phenotype. Genomic DNA from dead and alive mosquitoes was extracted using Livak method [28]. The amplification was performed using primers designed for *An*. *funestus s*.*s*. as previously described [20]. The PCR products were purified using the Qiaquick purification kit (QIAgen, Hilden, Germany) sequenced and then aligned using BioEdit. Sequences were aligned using ClustalW [34] and haplotype reconstruction and polymorphism analysis were done using DnaSP v5.10 [35]. After the selection of the best model, a maximum likelihood phylogenetic tree was generated for the VGSC haplotypes in Gounougou for dead and alive individuals to DDT and permethrin samples using Mega 5.2 [36].

### Genotyping of L119F-GSTe2 and A296S markers

The L119F-GSTe2 mutation shown to play a major role in DDT resistance in *An*. *funestus* was genotyped, using a TaqMan assay [11]. This was done using 50 field collected (F_0_) females to estimate the frequency of the 119F-GSTe2 mutation in Gounougou. It was also genotyped in 35 dead (susceptible) and 30 alive (resistant) F_1_ female mosquitoes after DDT exposure to assess its correlation with DDT resistance. In addition, this marker was also genotyped for mosquitoes collected in 2006 to assess the temporal frequency of this mutation in this location. The TaqMan reactions were performed as previously described [11] in a 10μl final volume containing 1xSensiMix (Bioline), 800nM of each primer and 200nM of each probe using the Agilent MX3005P machine.

Similarly, the A296S-RDL mutation conferring dieldrin resistance was also genotyped using a TaqMan assay [37] in 35 dead (susceptible) and 35 alive (resistant) F_1_ female mosquitoes after dieldrin exposure to assess its correlation with dieldrin resistance. 50 F_0_ females were also genotyped for the A296S mutation. In addition, this marker was also genotyped in 50 F_0_ females collected in 2006 as done for L119F-GSTe2 mutation.

## Results

### Field collection

A total of 402 F_0_ female mosquitoes collected resting indoor in 2012 were morphologically identified as belonging to *An*. *funestus* group. PCR species identification revealed that all mosquitoes were *An*. *funestus s*.*s*. apart from two individuals that were confirmed to be *An*. *leesoni* (also from *An*. *funestus* group). 3700 mosquitoes were generated from 302 F_1_ eggs batches reared successfully. For 2015, 165 F_1_ egg batches were obtained.

### Insecticides resistance profiles

**Fig 1:**
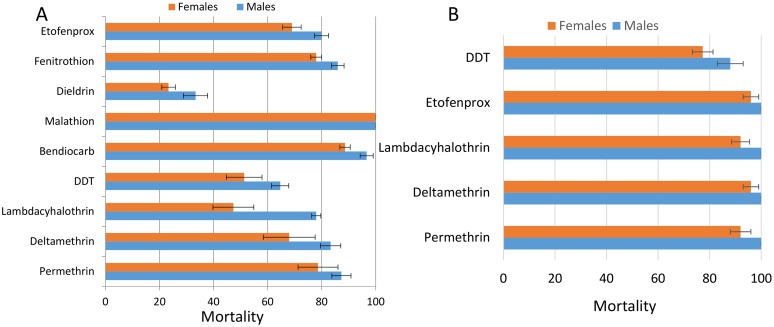
Susceptibility status of the *An*. *funestus* population from Gounougou in 2012; A) Mortality rates after 1hr exposure to insecticides; B) Mortality rate after PBO synergist assay. Error-bars represent the standard error (n = 4).

A total of 3410 mosquitoes from 2012 were tested to assess the resistance profile to nine insecticides. WHO bioassays carried out using F_1_ adults revealed that the *An*. *funestus* population from Gounougou (females and males) were resistant to both type I and type II pyrethroids (Fig 1A). The mosquitoes exhibited higher resistance towards the type II pyrethroids with a mortality rate of 68±5% for deltamethrin and 47.3±3% for lambda-cyhalothrin compared with mortality obtained from exposure with type I pyrethroid, permethrin (78.6±7%). This is similar to the patterns observed in East Africa [19] and southern Africa [37] but opposite to the situation in Benin, West Africa [20]. The population was also resistant to the pyrethroid derivative etofenprox with mortality rate of 69±3.6% for females.

For organochlorines, high resistance was observed with a mortality rate in the females of only 51±6.6% for DDT. An even higher resistance level was observed against dieldrin with only 23.3 ± 2.5% mortality in females (Fig 1A).

Resistance was observed against the carbamate bendiocarb but not at the levels for pyrethroids and DDT, with a mortality rate of 88.7±1.9% for females although males exhibited a higher mortality rate at 96.7±2.8% (Fig 1A).

In contrast to other insecticides, a full susceptibility was observed against the organophosphate malathion. A mortality rate of 78% was observed against the other organophosphate fenitrothion but this could be due to the exposure time of 1h which has since been changed to 2h for this insecticide (WHO, 2013).

#### PBO synergist assays

Pre-exposure of *An*. *funestus s*.*s*. to PBO significantly restored the susceptibility of this populations to type I and type II pyrethroids with mortality rates between 92 to 96% in females and 100% in males (Fig 1B). However, for DDT, a recovery of susceptibility was lower than for pyrethroids with mortality of 77.3% for females.

#### Increased resistance intensity between 2011 and 2015

**Fig 2:**
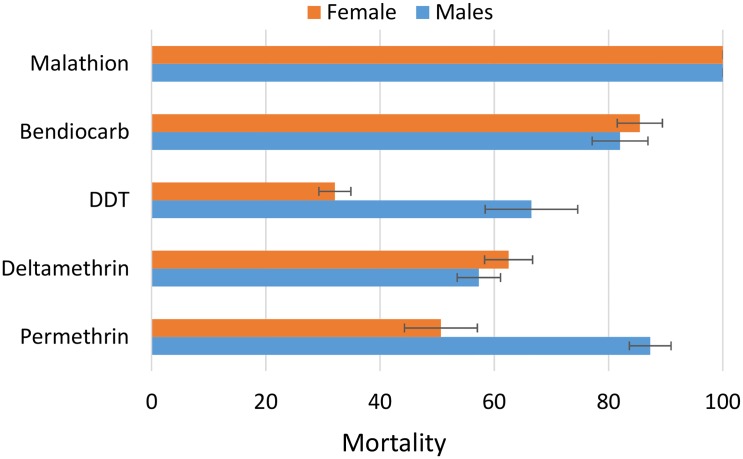
Susceptibility status of the *An*. *funestus* population from Gounougou in 2015. Error-bars represent the standard error (n = 4).

Assessment of the susceptibility levels of the Gounougou population in 2015 indicated an overall increase in resistance towards pyrethroids and DDT. For pyrethroids, the mortality rate for permethrin reduced from 78.6 to only 50.6% for females, and from 68 to 57.3% for deltamethrin (Fig 2). A similar increase in resistance is observed for DDT with a reduction in mortality rate from 51 to 32% (Fig 2). No major change was observed in resistance to bendiocarb (88.6% vs 82% for 2011 and 2015 respectively). In addition, the population still remains fully susceptible to the organophosphate malathion.

### Analysis of the VGSC polymorphism

**Fig 3:**
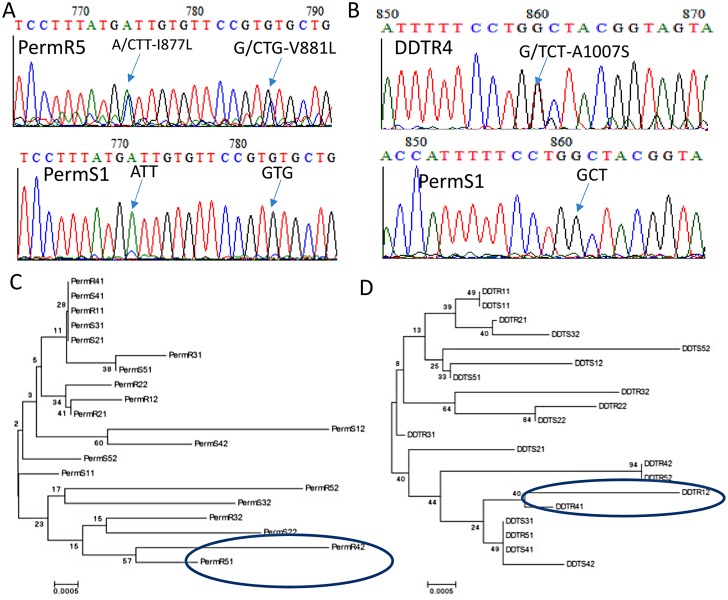
Analysis of the polymorphism of the voltage gated sodium channel (VGSC) gene: A) Sequencing traces showing the A877L and V881L mutations whereas B) shows the A1007S mutation. C) Maximum likelihood phylogenetic tree of VGSC fragment showing a lack of association between haplotypes and resistance phenotype to permethrin. B) is for DDT. R and S in the haplotype names stand for resistant and susceptible respectively.

A 924 bp portion of the VGSC gene spanning intron 19 and the entire exon 20 (207bp) was sequenced in a total of 20 mosquitoes from the 2012 collection, including 5 resistant and 5 susceptible mosquitoes each for permethrin and DDT, respectively. Overall, high polymorphism was observed for both resistant and susceptible samples for permethrin and DDT with a total of 38 substitutions detected (Table 1). Though no mutation was observed at the 1014 codon position, three other amino acid changes were detected: I877L (ATT to CTT), V881L (GTG to CTG) (Fig 3A) and the A1007S (GCT to TCT) (Fig 3B). The A1007S mutation was found in two DDT resistant samples and in one permethrin susceptible sample. The I877L and V881L mutations were found in two permethrin resistant samples.

**Table 1:** Summary statistics for polymorphism in the voltage-gated sodium channel gene in susceptible and resistant *An*. *funestus* from Gounougou. N, number of sequences (2n); S, number of polymorphic sites; h, number of haplotypes (haplotype diversity); π, nucleotide diversity (k = mean number of nucleotide differences); D and D*, Tajima’s and Fu and Li’s statistics; ns, not significant

Samples	N	S	π (k)	h (hd)	Syn	Nonsyn	D	D*
**Permethrin**								
**Resistant**	10	16	0.0054(5.0)	9(0.98)	0	2(I877L, V881L)	-0.52^ns^	-0.42^ns^
**Susceptible**	10	17	0.0049(4.5)	8(0.93)	2	1(A1007S)	-1.14^ns^	-0.99^ns^
**Total**	20	33	0.0052(4.8)	16(0.95)	2	3	-1.43^ns^	-1.42^ns^
**DDT**								
**Resistant**	10	14	0.0053(4.87)	9(0.978)	0	1(A1007S)	-0.38^ns^	-0.03^ns^
**Susceptible**	10	14	0.0043(4.00)	9(0.978)	0	0	-0.89^ns^	-0.96^ns^
**Total DDT**	20	28	0.0048(4.48)	16(0.97)	0	1	-1.18^ns^	-1.22^ns^
**Total all**	40	38	0.0051(4.7)	28(0.98)	2	3	-1.71ns	-2.48ns

#### Correlation between resistance phenotype and haplotype diversity

Analysis of the maximum likelihood phylogenetic tree of the VGSC sequences did not reveal an association between VGSC polymorphism and pyrethroid (Fig 3C) or DDT (Fig 3D) resistance. This lack of correlation was further supported by the maximum likelihood haplotype phylogenetic tree which also did not show a clustering of haplotypes according to their resistance phenotype. In addition, the relatively high polymorphism of the VGSC is shown by the high number of haplotypes and the absence of a predominant haplotypes for permethrin samples. This suggests that the VGSC is probably evolving neutrally contrary to what is expected if knockdown resistance was playing a significant role in the observed pyrethroid and DDT resistance. The absence of selection is further supported by estimates of Tajima D and Fu and Li D* which are all non-significant in Gounougou (Table 1). However, the mosquitoes carrying the detected amino acid changes clustered together suggesting that a possible low level selection could be ongoing in the population.

### Role of the L119F-GSTe2 mutation in DDT resistance in Gounougou

The TaqMan genotyping of the L119F-GSTe2 in F_0_ field collected females revealed a high frequency of 119F resistant allele in Gounougou population (52%) in correlation with the DDT resistance observed in this location (Fig 1A). Furthermore, the genotyping of the L119F-GSTe2 mutation in 35 susceptible and 35 DDT resistant mosquitoes revealed that the frequencies of the resistant allele was 72.5% in DDT resistant mosquitoes but only 10% in the susceptible allele. A significant association was observed between the L119F mutation and the DDT resistance with an odds ratio of 5.74 (P < 0.0001) confirming a previous observation [11].

**Fig 4:**
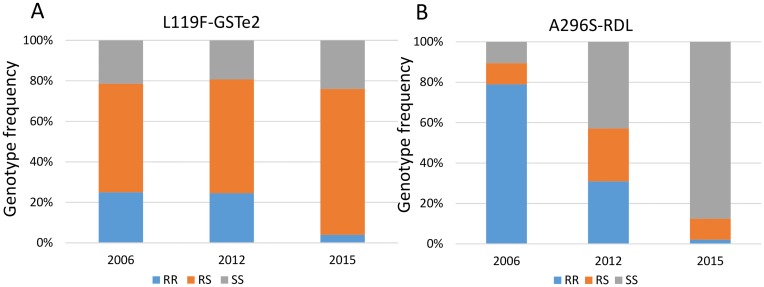
Temporal distribution of resistance markers in Gounougou. A) Frequency of the three genotypes of the L119F-GSTe2 conferring DDT resistance between 2006, 2012 and 2015; B) is for A296S-RDL mutation conferring dieldrin resistance.

The temporal distribution of the 119F resistance allele was assessed in Gounougou by also genotyping a sample collected in December 2006 and January 2015 in the same location. This indicated that the frequency of the 119F was relatively stable in Gounougou with the same frequency of 52% also observed in 2006 than 2012 although a slight drop was observed in 2015 to 40% due to a reduction of the homozygote resistant genotype 119F/F (Fig 4A).

### Detection of the A296S RDL mutation correlation with dieldrin resistance

The three genotypes of the A296S RDL mutation were successfully detected in the *An*. *funestus* population in Gounougou. The frequency of the resistant 296S allele in Gounougou was 45% correlating with the observed dieldrin resistance in this location with only 24% mortality (Fig 1). Genotyping of the A296S mutation in 30 dieldrin resistant and 30 susceptible mosquitoes to assess the correlation between the A296S RDL mutation and the resistance phenotype revealed that dieldrin resistant mosquitoes showed a higher frequency (90%) of the resistant allele 296S RDL compared to the susceptible mosquitoes (40%).

The temporal distribution of the 296S resistance allele was assessed in Gounougou by genotyping a sample collected in December 2006 in the same location. In contrast to L119F-GSTe2, a significant difference was observed between the three collections with a marked continuous reduction of the frequency of the resistant allele from 80% in 2006 to 40% in 2012 and to only 14.6% in 2015 (Fig 4A). This temporal variation is also observed in the distribution of the three RDL resistance genotypes from 2006 to 2015 (Fig 4B).

## Discussion

The WHO global plan of insecticide resistance management (WHO, 2012) highlights the necessity to detect and monitor the development of insecticide resistance and characterise the underlying resistance mechanisms in order to maintain the successes in reduction of malaria cases across Africa. In this study, the first survey of the insecticide resistance patterns of the major malaria vector *An*. *funestus s*.*s*. in Central Africa has revealed resistance to multiple insecticides, likely driven by metabolic resistance and target-site resistance mechanisms posing a challenge that control programs should take into consideration.

### 1-Multiple resistance is a challenge to resistance management

The *An*. *funestus* population from Gounougou exhibited resistance towards three of the four main insecticide classes, notably against pyrethroids the only class recommended for LLINs. The overall resistance profile of this Central African population of *An*. *funestus* is similar to resistance patterns previously reported in West Africa, notably in Benin where a population of *An*. *funestus s*.*s*. was reported to be resistant to organochlorines, pyrethroids and carbamates [20]. Similar observations were made in southern Ghana in 2008 [21] and in 2014 [38] suggesting that multiple resistance could be already widespread in populations of this major vector from West to Central Africa. However, this pattern of resistance form Cameroonian *An*. *funestus s*.*s*. is different to that observed in East Africa (Kenya and Uganda) where no carbamate resistance is observed [19], and is also different to the situation in southern Africa where report of DDT resistance is still limited [37]. Because previous studies have highlighted significant level of gene flow among populations of these Central and West African regions [39, 40], it is possible that the similarity of the resistance profile between these regions could stem from the spread of resistance genes between populations through migration. However, some differences were observed between this Central African population and that from Benin in West Africa particularly for the level of DDT and dieldrin resistance. Indeed, in Benin, the *An*. *funestus* populations exhibited a very high level of resistance with no mortality at all observed after 1h exposure to DDT [20] whereas in Gounougou the mortality was 51% in 2012. However, the Gounougou population showed a high level of dieldrin resistance (23% mortality) whereas the Benin population was only moderately resistant to this insecticide (93.3% mortality) [20]. This difference also suggests that even if gene flow can explain similarity of resistance profiles, local selection forces are also shaping the patterns of resistance. These selection forces in Gounougou could come from agricultural use of insecticides notably in cotton fields where both DDT and pyrethroids are used by farmers [26]. The scale up of insecticide-based interventions could also be driving the rise of this resistance as a preliminary assessment of susceptibility of the *An*. *funestus* from Gounougou in 2006 had revealed that it was still fully susceptible to permethrin and deltamethrin (100% mortality after 1h and 30min) (C. Wondji and M. Chouaibou; Unpublished data). Similarly, a survey performed on *An*. *gambiae* in 2006 in the same location indicated only a moderate resistance to pyrethroids (89% mortality to deltamethrin) and DDT (95% mortality) and full susceptibility to carbamates and organophosphates [26] showing that resistance has steadily risen in recent years coinciding with scale up of LLINs and IRS. This rise of resistance was confirmed when comparing the 2012 and 2015 samples as recently shown in a southern African population of *An*. *funestus* from southern Malawi [37]. This rise of resistance points to a worsening of the resistance problem in malaria vectors [41] and calls for urgent action to be taken to tackle this serious threat.

### 2-Metabolic resistance is predominant in the pyrethroids/DDT resistance

An exploration of the potential mechanisms driving this multiple resistance in the *An*. *funestus* population from Gounougou suggested that metabolic resistance is probably playing a predominant role. This is supported by the PBO synergist assays which showed a high recovery of susceptibility for pyrethroids. This high recovery after PBO exposure is similar to the profiles obtained with other *An*. *funestus* samples across the continent for which cytochrome P450s have been demonstrated to drive resistance to pyrethroids, including genes such as the duplicated *CYP6P9a* and *CYP6P9b* [23, 25], or *CYP6M7* [24]. It is likely that P450s are also the main pyrethroid resistance in Gounougou although future studies will help to establish this and detect whether the main resistance genes are the same as in southern Africa. The reduced level of recovery of susceptibility to DDT after PBO exposure (23% still alive) than for pyrethroids (4–8% alive) suggests other metabolic resistance genes in play, other than the cytochrome P450s, as previously reported in Benin [20]. The absence of the L1014F/S mutations and lack of correlation between VGSC haplotype diversity and DDT resistance, suggests that DDT resistance is mainly conferred by the over-expression of GST genes such as *GSTe2* coupled with the presence of the L119F-GSTe2 mutation as previously established [11]. The high frequency of the 119F-GSTe2 allele supports the role of this gene in DDT resistance although other genes could also be involved. The high frequency of 119F-GSTe2 resistant allele in Cameroon is comparable to the situation in Benin, but less comparable to Uganda where the frequency is low despite significant DDT resistance. It is also different to southern Africa where this mutation is completely absent despite recent reports of DDT resistance [15, 37]. These strong heterogeneities in L119F frequencies suggest the presence of different mechanisms responsible for the DDT resistance in this species across Africa and also point to the presence of barriers to gene flow between populations from different regions.

Although no *kdr* mutation was detected at the 1014 codon, sequencing of a portion of the VGSC identified three amino acid changes with one, A1007S, found in two DDT resistant samples and also located near the 1014 codon in the S6 hydrophobic segment of domain II of the VGSC gene. From these 3 amino acid changes, only the A1007S is a replacement between two different types of amino acids, an aliphatic to a neutral, thus could have a greater impact on activity of the sodium channel than the other two mutations of I877L and V881L which are replacements between amino acids of the same type. This will be similar to what has been observed in other species such as *Aedes aegypti* where *kdr* mutations conferring pyrethroid resistance are observed at codon 1011 and 1016 [42] or the L932F in *Culex quinquefasciatus* [43]. Another such mutation, the F1021C, was also recently detected at a low frequency in an *An*. *funestus* populations from Uganda [19] suggesting that knockdown resistance could be present in this species. Further studies will help establish the role of these mutations in the pyrethroid/DDT resistance. Monitoring these VGSC mutations is important in order to spot such resistance when it is still at early stage, and when it is easier to contain it through suitable resistance management strategies [2].

The significant differences observed in the temporal analysis of the frequency of the L119F-GSTe2 and of the A296S-RDL alleles could indicate that these two mutations are under two different evolution dynamics. The relative stability of 119F-GSTe2 suggests a continued selection pressure from DDT in line with the agricultural use of this insecticide in this location [26]. However, further studies will help to understand why a slight drop of the frequency of the 119F-GSte2 allele was observed in 2015 despite the increased DDT resistance. This could be due to possible sample bias or the increased role of alternative DDT resistance mechanisms. In the case of the A296S-RDL mutation, the continuous decrease in the frequency of this allele could suggest not only a reduced selective pressure from dieldrin, but also a possible fitness cost associated with bearing this allele. This is in line with a recent evidence in *An*. *gambiae* population showing that mosquitoes homozygotes for the 296S resistant allele are less competitive in mating than mosquitoes bearing the susceptible allele [44].

## Conclusion

This study reports the detection of a multiple resistance in a Central African populations of the major malaria vector *An*. *funestus* highlighting the challenges that control programs face in maintaining the continued effectiveness of current insecticide-based control intervention. Further studies should be carried out to assess the distribution of such resistance and efforts put in place to manage it before it leads to control failure.
